# Correction: Chiral poly(aza-norbornene) derivatives with tunable tacticity and living ROMP capability

**DOI:** 10.1039/d6sc90040b

**Published:** 2026-02-12

**Authors:** Jing Bai, Yu Wang, Yisong Wang, Na Zhang, Xiaoyang Wang, Yan Xu, Wei You

**Affiliations:** a Beijing National Laboratory for Molecular Sciences, CAS Key Laboratory of Engineering Plastics, Institute of Chemistry, Chinese Academy of Sciences Beijing 100190 China weiyou@iccas.ac.cn ywang507@iccas.ac.cn; b University of Chinese Academy of Sciences Beijing 100049 China; c Beijing National Laboratory of Molecular Sciences (BNLMS), Key Laboratory of Bioorganic Chemistry and Molecular Engineering of Ministry of Education, College of Chemistry and Molecular Engineering, Peking University Beijing 100871 China yanx@pku.edu.cn

## Abstract

Correction for ‘Chiral poly(aza-norbornene) derivatives with tunable tacticity and living ROMP capability’ by Jing Bai *et al.*, *Chem. Sci.*, 2026, **17**, 2233–2244, https://doi.org/10.1039/d5sc07016c.

The authors regret that the specific rotation values reported in the paper for the compounds P-R4-Ru3 and P-R4-Ru5 were mistakenly switched in the discussion section. The penultimate sentence of the second last paragraph of page 2235 should read: “The specific rotation of P-R4-Ru3 was determined to be −29° mL g^−1^ dm^−1^ (5 mg mL^−1^ in chloroform), while that of P-R4-Ru5 was +30° mL g^−1^ dm^−1^.”

The Royal Society of Chemistry apologises for these errors and any consequent inconvenience to authors and readers.

